# Determinants of cerebral collateral circulation in acute ischemic stroke due to large vessel occlusion

**DOI:** 10.3389/fneur.2023.1181001

**Published:** 2023-05-17

**Authors:** Martina Sperti, Francesco Arba, Amedeo Acerbi, Giorgio Busto, Enrico Fainardi, Cristina Sarti

**Affiliations:** ^1^Department of Neurofarba, University of Florence, Florence, Italy; ^2^Stroke Unit, Careggi University Hospital, Florence, Italy; ^3^Neuroradiology, Careggi University Hospital, Florence, Italy

**Keywords:** ischemic stroke, large vessels occlusion, anterior circulation, cerebral collateral circles, cerebral collateral vessels, leptomeningeal vessels, multiphase CTA, stroke prognosis

## Abstract

**Introduction:**

Cerebral collateral circulation has a central role in ischemic stroke pathophysiology, and it is considered to correlate with infarct size, the success of reperfusion therapies, and clinical outcomes. Our aim was to study the factors influencing the development of collaterals in patients with acute ischemic stroke eligible for endovascular treatment.

**Materials and methods:**

We enrolled patients with acute ischemic stroke and large vessel occlusion of anterior circulation potentially eligible for endovascular treatment. Included patients performed multiphase CT angiography to assess collaterals that were graded by the Menon Grading Score. We investigated the associations between clinical factors and collaterals and tested independent associations with logistic (good vs. poor collaterals) and ordinal (collateral grade grouped, Menon 0–2, 3, 4–5) regression analysis adjusting for age, sex, stroke severity, and onset to CT time (OCTT).

**Results:**

We included 520 patients, the mean age was 75 (±13.6) years, 215 (41%) were men, and the median (IQR) NIHSS was 17 (11–22). Good collaterals were present in 323 (62%) patients and were associated with lower NIHSS (median 16 vs. 18; *p* < 0.001) and left hemisphere involvement (60% vs. 45%; *p* < 0.001), whereas previous stroke/TIA was more frequent in patients with poor collaterals (17 vs. 26%; *p* = 0.014). These results were confirmed in both logistic and ordinal regression analyses where good collaterals were associated with lower NIHSS (OR = 0.94; 95% CI = 0.91–0.96; cOR = 0.95; 95% CI = 0.92–0.97, respectively) and left hemisphere stroke (OR = 2.24; 95% CI = 1.52–3.28; cOR = 2.11; 95% CI = 1.46–3.05, respectively), while previous stroke/TIA was associated with poor collaterals (OR = 0.57; 95% CI = 0.36–0.90; cOR = 0.61; 95% CI = 0.40–0.94, respectively). Vascular risk factors, demographics, and pre-stroke treatments did not influence the collateral score.

**Discussion:**

The results of our study suggest that risk factors and demographics do not influence the development of collateral circles, except for a negative relation with previous ischemic events. We confirm an already reported observation of a possible protective effect of collaterals on tissue damage assuming NIHSS as its surrogate. The association between left hemispheric stroke and better collaterals deserves to be further explored. Further efforts are needed to identify the factors that favor the development of collaterals.

## 1. Introduction

Endovascular treatment (EVT) of acute ischemic stroke (AIS) proved to be dramatically effective in reducing the disability and mortality of patients. (1) Reperfusion of the ischemic but still viable tissue, namely ischemic penumbra, and reducing the growth of the irreversibly ischemic tissue (i.e., ischemic core) are the objectives of acute stroke therapy. Several metabolic and genetic factors as well as pre-existing characteristics of the cerebral tissue may influence the extent of both the ischemic penumbra and ischemic core (2). In previous studies, leptomeningeal cerebral collaterals (CC) have been associated with lower infarct volume (3) and better clinical outcomes (4).

Cerebral collaterals (CC) have been classified into primary collaterals, which provide fast support to the ischemic area, and secondary collaterals, also constituted by leptomeningeal vessels, which require time to be supportive for the ischemic brain tissue. Pathophysiological factors leading to the development of CC are uncertain (5). The role of age (3, 6, 7), sex, site of occlusion, and cardiovascular risk factors, such as dyslipidemia and statin use as determinants of CC, has been investigated in several studies (3, 6–10). However, the reported results were often conflicting and not conclusive. Understanding determinants of CC might help to develop target therapeutic strategies to increase ischemic brain blood supply in the acute phase of stroke and consequently to improve the early prognosis of patients with AIS (8).

In patients with AIS eligible for EVT, we explored the associations between clinical factors and CC grade.

## 2. Materials and methods

We conducted a single-center observational retrospective study. We enrolled patients admitted for AIS potentially eligible for EVT at the Stroke Unit of a Comprehensive Stroke Center University Hospital (Florence, Italy) between 2017 and 2022.

We included patients with middle cerebral artery (MCA) occlusion (M1 and/or M2) with (tandem occlusion) or without occlusion of the internal carotid artery (ICA). Only patients with available pre-treatment multiphase CT angiography (mCTA) were included. We excluded patients with hemorrhagic stroke, patients with vertebrobasilar ischemic stroke, and patients with ischemic stroke due to isolated ICA, M3 segment of MCA, or anterior cerebral artery occlusion.

We collected the following clinical data: demographic, anamnestic [hypertension, diabetes, dyslipidemia, smoking habit, previous stroke or transient ischemic attack (TIA), and atrial fibrillation], current medication at the time of stroke (i.e., statin, antithrombotic/anticoagulant, or antihypertensive drugs), and at the time of admission using the National Institutes of Health Stroke Scale (NIHSS). We also collected neuroradiological imaging data such as time from stroke onset (or last time seen well) to brain imaging and side of the involved cerebral hemisphere.

### 2.1. Imaging study protocol

An mCTA with a 0.625 mm slice thickness in a three-time-point acquisition was performed from the aortic arch to the cranial vertex (phase 1) and from the skull base to the vertex (phase 2 and phase 3 after each 8 s delay) for 150–260 mAs radiation dose. A total of 50 ml of iodinated contrast was injected at 5 ml/s, followed by a 30-ml normal saline chase. mCTA provided identification of large vessel occlusion (LVO) and evaluation of CC according to the Menon Grading Score.

**Figure 1:**
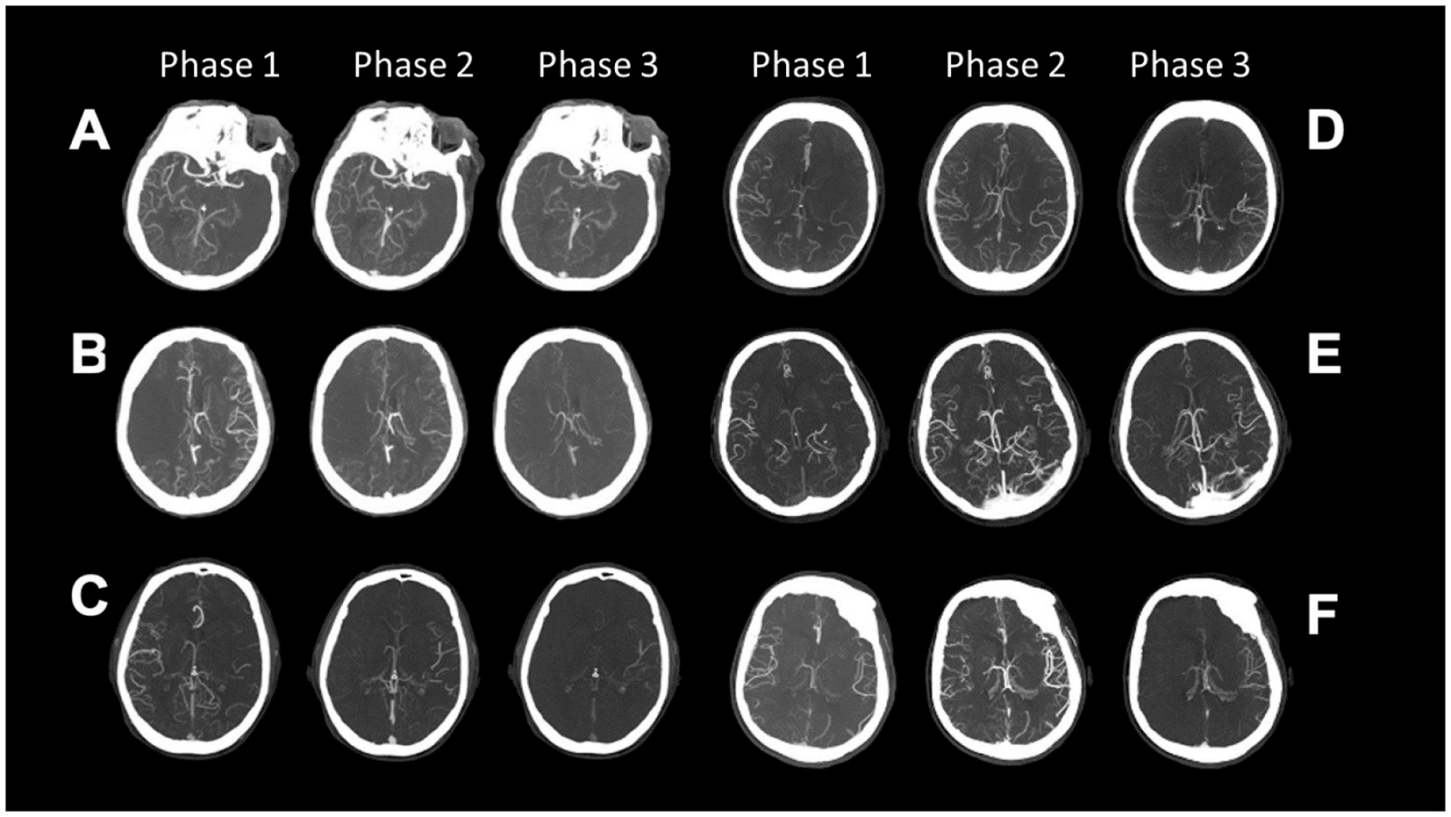
Collaterals on three phases of multiphase CT Angiography classified according to the Menon Grading Score **(A–F)** is Grade 0–5; (Table 1).

The latter was defined by two neuroradiologists (GB and EF) blinded to clinical data and classified according to a six-point ordinal scale described by Menon et al. (11) as reported in Figure 1 and Table 1 (11).

**Table 1:** Menon grading score.

**A**	**Grade 0. When compared with the asymptomatic contralateral hemisphere, there are no vessels visible in any phase within the ischemic vascular territory**
B	**Grade 1**. When compared with the asymptomatic contralateral hemisphere, there are just a few vessels visible in any phase within the occluded vascular territory
C	**Grade 2**. When compared with the asymptomatic contralateral hemisphere, there is a delay of two phases in filling in of peripheral vessels and decreased prominence and extent or a one-phase delay, and some ischemic regions with no vessels
D	**Grade 3**. When compared with the asymptomatic contralateral hemisphere, there is a delay of two phases in filling in of peripheral vessels or there is a one-phase delay and a significantly reduced number of vessels in the ischemic territory
E	**Grade 4**. When compared with the asymptomatic contralateral hemisphere, there is a delay of one phase in filling in of peripheral vessels, but prominence and extent are the same
F	**Grade 5**. When compared with the asymptomatic contralateral hemisphere, there is no delay and normal or increased prominence of pial vessels/normal extent within the ischemic territory in the symptomatic hemisphere

Letters (A–F) refers to Figure 1.

Cerebral collaterals (CCs) were then categorized into good (0–2), intermediate (3), and poor (4, 5). The predictive ability of clinical outcomes was categorized on a cutoff of 0–3 as poor and 4–5 as moderate-to-good CC.

### 2.2. Statistical analysis

To explore putative factors associated with CCs, we performed two separate analyses. In the first analysis, we divided the population into two groups: good collaterals (Menon grades 4–5) and poor collaterals (Menon grades 0–3). We described the general characteristics of the population and used Student's *t*-test, Mann–Whitney *U*-test, and Pearson's chi-square test, as appropriate, to test differences between groups. We examined univariate associations with a *p*-value of < 0.1 in a multivariable logistic regression model adjusting for age, sex, NIHSS, and onset to CT time (OCTT). In the second analysis, we categorized the population into three groups: poor collaterals (Menon grades 0–2), intermediate collaterals (Menon grade 3), and good collaterals (Menon grades 4–5). We examined univariate associations with a *p*-value of < 0.1 in a multivariable ordinal regression model adjusting for age, sex, NIHSS, and OCTT. For both multivariable analyses, we considered a *p*-value of <0.05 statistically significant. Statistical analysis was performed using SPSS for Windows (version 24.0; SPSS, IBM Corp., Armonk, NY). The data that support the findings of this study are available from the corresponding author upon reasonable request.

## 3. Results

In the observation period, we included 520 patients with AIS, eligible for EVT. The general characteristics of the study population are shown in Table 2. The mean age was 75 (±13.6) years, and 215 (41%) were men. Median (IQR) NIHSS was 17 (11–22), and median (IQR) OCTT was 240 min (130–420). In total, 45 (8%) patients were treated with intravenous thrombolysis, 226 (44%) with EVT, 220 (42%) with both, and 29 (6%) were not treated with reperfusion therapy.

**Table 2:** General characteristics of the study population according to poor and good collaterals.

	**Total (*n=* 520)**	**Poor collaterals (Menon 0-3) *n =* 197**	**Good collaterals (Menon 4-5) *n =* 323**	**P (univariate)**
**Age mean (SD)**	75.04 (14)	75.5 (13)	74.75 (14)	0.528
**Sex, M (n, %)**	215 (41)	87 (44)	128 (40)	0.308
**NIHSS (median, IQR)**	17 (11–22)	18 (14–23)	16 (10–21)	**<0.001**
**OCTT, min (median, IQR)**	240 (130–420)	210 (120–385)	260 (140–440)	**0.019**
**Left hemisphere**	283 (54)	89 (45)	194 (60)	**<0.001**
**Hypertension**	405 (79)	155 (80)	250 (78)	0.667
**Diabetes**	160 (32)	67 (36)	93 (30)	0.178
**Dyslipidemia**	224 (44)	89 (46)	135 (42)	0.369
**Atrial fibrillation**	185 (36)	81 (42)	104 (32)	**0.036**
**Past stroke/TIA**	106 (21)	51 (26)	55 (17)	**0.014**
**Past smoking**	134 (34)	56 (36)	78 (32)	0.450
**Current smoking**	90 (19)	33 (19)	57 (20)	0.783
**Statins use**	164 (32)	62 (32)	102 (32)	0.996
**Antihypertensives use**	366 (71)	193 (71)	227 (71)	0.891
**Antiplatelets use**	161 (31)	62 (32)	99 (31)	0.803
**Anticoagulants**	111 (22)	45 (23)	66 (21)	0.489

SD, standard deviation; IQR, interquartile range; NIHSS, National Institutes of Health Stroke Scale; OCTT, onset-to-CT time; TIA, transient ischemic attack. Data are numbers (%) unless otherwise stated. Bold values are statistically significant *p* values.

The distribution of vessel occlusions was as follows: 287 (55%) M1, 92 (18%) M2, 92 (18%) M1–M2, 49 (9%) tandem occlusion, and 283 (54%) patients had a stroke in the left hemisphere.

**Figure 2:**
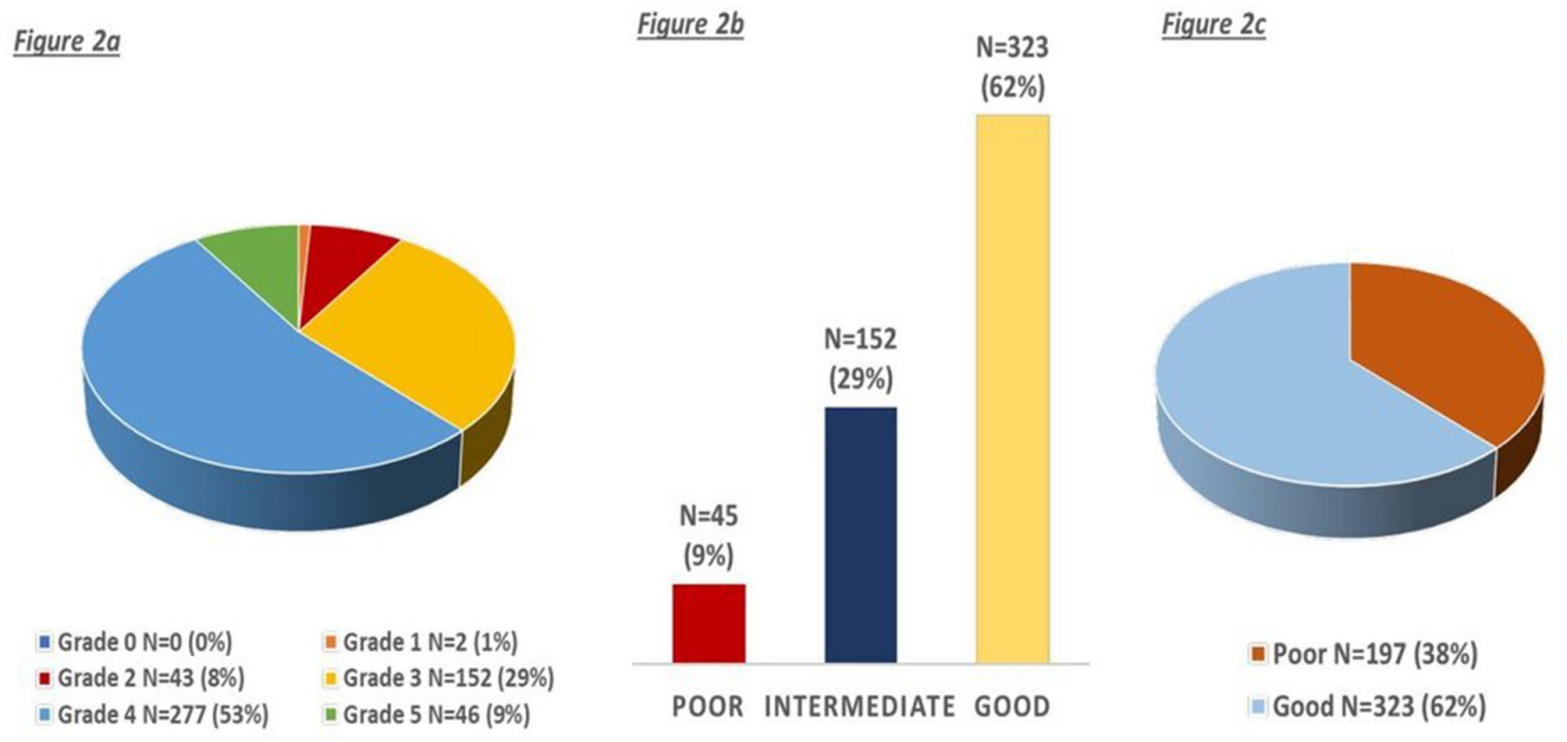
**(a–c)** Distribution of collaterals according to the ordinal and three-point menon grading score.

The distribution of CC status is shown in Figures 2a–c; most of the patients had good CC defined as Menon grades 4–5 (*n* = 323, 62%).

Patients with good CC had lower NIHSS (median 16 vs. 18; *p* < 0.001) and longer OCTT (median 260 min vs. 210 min; *p* = 0.019), had more frequent ischemia in the left hemisphere (60 vs. 45%; *p* < 0.001) but less frequently atrial fibrillation (32 vs. 42%; *p* = 0.036) and past stroke/TIA (17 vs. 26%; *p* = 0.014). There were no other differences in the general characteristics of the population. In the logistic regression analysis (Table 3), NIHSS (OR = 0.94; 95% CI = 0.91–0.96) and left hemisphere stroke (OR = 2.24; 95% CI = 1.52–3.28) were associated with good collaterals, whereas previous stroke/TIA was associated with reduced odds to have good collaterals (OR = 0.57; 95% CI = 0.36–0.90).

**Table 3:** Multivariate analysis of collaterals graded by the dichotomic Menon scale.

**Variable**	**OR**	**95% CI**
NIHSS^1^	0.94	0.91–0.96
OCTT, min^2^	1.00	0.99–1.02
Past stroke/TIA^3^	0.57	0.36– 0.90
Left hemisphere^3^	2.24	1.52–3.28
Atrial Fibrillation^3^	0.69	0.47–1.03

^1^Analysis adjusted for age, sex, and onset to CT time. ^2^Analysis adjusted for age, sex, and NIHSS. ^3^Analysis adjusted for age, sex, NIHSS, and onset to CT time.

**Figure 3:**
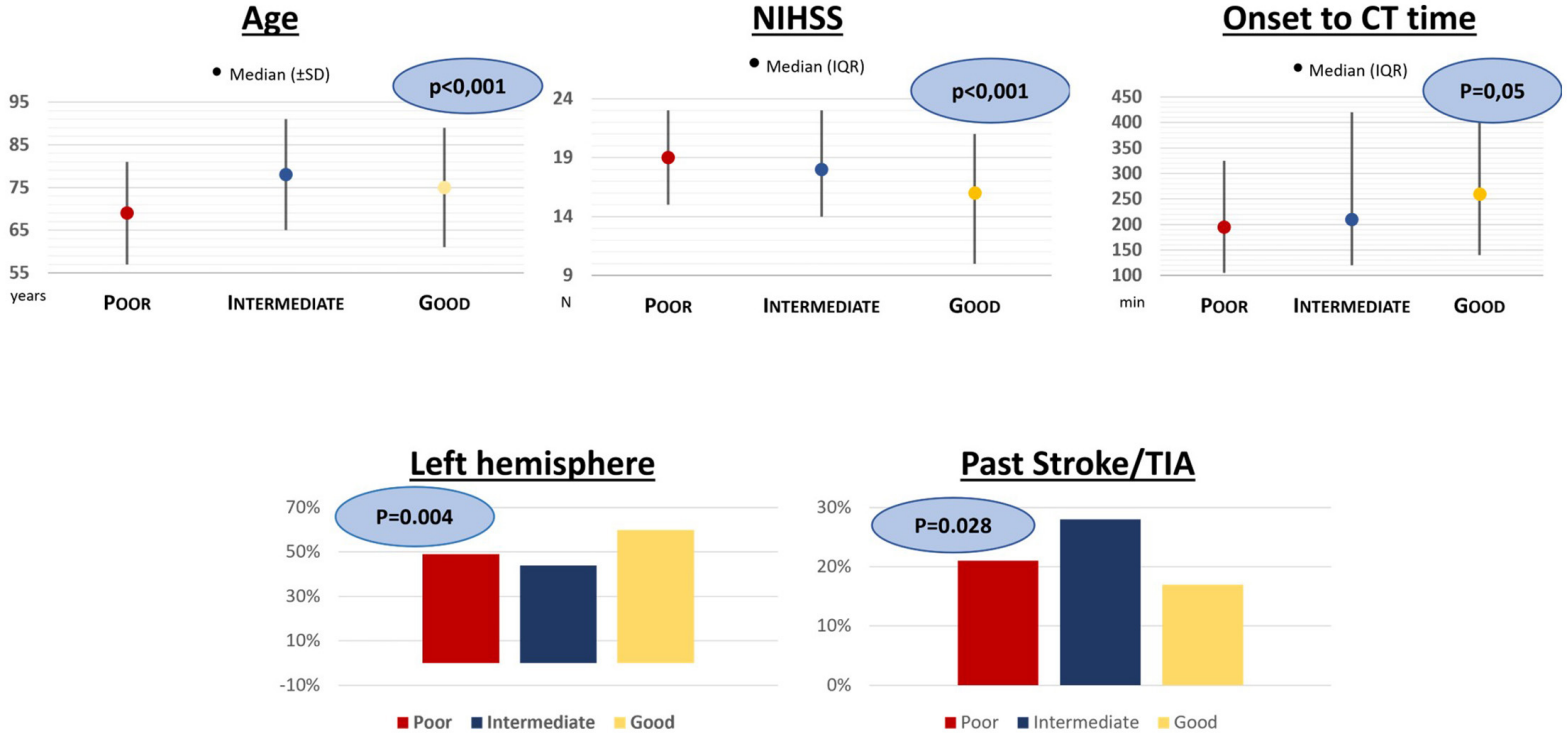
Results of univariate analysis for patients divided into three groups according to the Menon Grading Score.

Considering the grouped Menon grade, 45 (9%) had poor collaterals, 152 (29%) had intermediate collaterals, and 323 (62%) had good collaterals (Figure 2b). We observed a trend toward higher age (mean 69, 78, 75; *p* < 0.001), lower NIHSS (median 19, 18, 16; *p* < 0.001), and higher OCTT (195, 210, 260; *p* = 0.05) for poor, intermediate, and good collaterals, respectively. Similarly, patients with left hemisphere stroke (49, 44, 60%; *p* = 0.004) had more frequently good collaterals, whereas with previous stroke/TIA (21%, 28%, 17%; *p* = 0.028) they had more frequently poor collaterals (Figure 3). In ordinal regression analysis (Table 4), NIHSS (cOR = 0.95; 95% CI = 0.92–0.97) and left hemisphere stroke (cOR = 2.11; 95% CI = 1.46–3.05) were associated with better odds to have good collaterals as opposed to previous stroke/TIA, which was associated with worse collaterals (cOR = 0.61; 95% CI = 0.40–0.94).

**Table 4:** Multivariate analysis of collaterals graded by a three-point Menon scale.

**Variable**	**cOR**	**95% CI**
Age^1^	1.01	0.99–1.02
NIHSS^2^	0.95	0.92–0.97
OCTT, min^3^	1.001	1.000–1.002
Past stroke/TIA^4^	0.61	0.40–0.94
Left hemisphere^4^	2.11	1.46–3.05
Diabetes^4^	0.74	0.50–1.10

NIHSS, National Institutes of Health Stroke Scale; OCTT, onset to CT time; TIA, transient ischemic attack. ^1^Analysis adjusted for sex, NIHSS, and onset to CT time. ^2^Analysis adjusted for age, sex, and onset to CT time. ^3^Analysis adjusted for age, sex, and NIHSS. ^4^Analysis adjusted for age, sex, NIHSS, and onset to CT time.

## 4. Discussion

From their first description (5), CCs have been increasingly studied and found to be associated with final infarct size and clinical outcomes after reperfusion therapy (1, 6, 13). The conceptual evolution from “time window” to “tissue window” for acute reperfusion therapy led to the investigation of the role of pre-treatment CC in the selection of patients eligible for therapies.

As previous studies were inconclusive and discordant, the aim of our study was to further explore determinants of CC that could be of help in better understanding their pathophysiology. The next step would have been to plan strategies that can increase CC with the final goal to reduce the extent of the ischemic lesion and widen the therapeutic window. Our study failed to find a relation between vascular risk factors or demographic factors and CC development. Indeed, main modifiable and non-modifiable risk factors such as age, hypertension, diabetes, smoking habit, or dyslipidemia do not seem to influence the development of CC, apart from past stroke/TIA, which was found to be negatively related to CC. The latter is one of the most frequently investigated risk factors in the literature (6, 9, 15–18) again with inconclusive results. A recent study found that previous stroke is associated with slower progression of cerebral infarct, and the authors assume that this could be due to cerebral ischemic preconditioning, an adaptive phenomenon in which brief exposure to ischemia and reperfusion markedly enhances the resilience of the brain to subsequent ischemic insults, inducing arteriogenesis and collateral growth (18). Indeed, the idea that an ischemic insult could reduce infarct growth was explored in preclinical and clinical studies, showing that remote ischemic conditioning could be a strategy for the treatment of acute ischemic stroke. Improvement of cardiac function, increase in CC, the protection of neurovascular units, the formation of gas molecules, and the effect on the function of vascular endothelial cells and the nervous system are among the hypothesized mechanisms (19). Our results are not in line with this hypothesis. However, a previous randomized controlled trial showed an increased risk of symptomatic intracranial hemorrhage in patients treated with r-TPA and CT signs of an old infarct explaining it with the concept of “brain frailty,” and they also demonstrated that this is independently related to poor functional outcome (20). These results were then confirmed by subsequent studies mainly focused on small vessel diseases, showing that “brain frailty”-related CT elements have a dose-dependent relationship with poor collaterals and further led to poor prognosis (21, 22). Moreover, a recent study evidenced that those elements combined with collaterals have a better value to predict the prognosis of patients with acute large artery atherosclerotic stroke (23). These findings led us to hypothesize that all of these elements could be related to determining brain plasticity impairment and consequently the capability of the brain to develop collateral vessels. This points out that it could be worth having more details on the type, site, and size of previous stroke with a properly designed study to better understand the direction of this relationship that could be of help in making therapeutic decisions.

In our study, we also explored the possibility that antihypertensive drugs such as alpha-lithics could reduce the vascular tone and influence CCs but we did not find any association. The same results were obtained considering other pre-stroke treatments (i.e., statins, anticoagulants, or antiplatelets). Of note, the study was not powered to explore such a point so these findings have to be considered as results generating hypothesis.

In keeping with previous studies, we found that lower stroke severity, assessed with NIHSS, was associated with better collateral status (3, 6). Although with some limits due to the ischemic side, higher NIHSS reflects larger ischemic tissue volume (8). Given that CC status has been previously associated with the extent of ischemic core (3), NIHSS may represent a surrogate of it (24). The association of NIHSS with CC seems consistent across studies, and we found a similar magnitude of the effect of NIHSS on collateral status compared with other studies.

A recent study found that tissue fate depends on collateral blood flow and that the natural evolution of ischemic injury is irrespective of the time of assessment of the penumbra (25). Similarly, we found that longer OCTT was associated with better CC; however, this result may suffer from a selection bias, as we included in our study patients potentially eligible for EVT, excluding those not eligible (e.g., established large infarct at non-contrast CT, more than 24 h after stroke onset, pre-existing severe disability). However, this result may also reflect that in patients eligible for EVT, good CC may be useful to treat patients in the extended time window, as previously suggested (11, 26). The association between time from stroke onset and CC deserves further investigation to explore whether CC may reflect the tissue viability also in late arrivals.

To the best of our knowledge, the role of the ischemic side in relation to CC has never been investigated. We found that acute ischemia on the left hemisphere was independently associated with better collateral status. This could be explained assuming that a dominant hemisphere has to be more preserved and also requires a higher blood support for its elevated metabolic activity. A recent study with functional magnetic resonance on stroke patients showed that ischemia in the dominant hemisphere was associated with lower activation of the supplementary motor area on neural network reorganization, suggesting a difference in lateralization of this specific process in brain ischemia (27). Moreover, a previous study found a predilection for cerebrovascular disease due to large vessel occlusion at the left side possibly related to greater hemodynamic stress and intimal damage in the left carotid artery; further studies could be conducted in order to investigate whether these hemodynamic differences would also explain a different CC recruitment in two hemispheres (28, 29). A study conducted on infants showed that the left hemisphere has greater metabolic demands than the right and higher neonatal cerebral blood flow velocity on the left hemisphere is positively related to neurobehavioral maturation (30). Our findings about side and CC seem to suggest a better capacity of the dominant hemisphere in the recruitment of CC. This result deserves further examination, possibly with functional magnetic resonance studies.

Our study has certain limitations. First, the retrospective design did not allow a causal link between factors and CC; rather, we described associations that could be explored and confirmed in future prospective studies. Again, data were collected from a single academic center with a hub function for EVT in a large Metropolitan area; thus, we cannot exclude a selection bias toward higher stroke severity in our cohort. Furthermore, protocols for eligible patients for EVT may slightly differ from clinical trials and guidelines, as our population represents a real-world sample of stroke patients. Future studies should have a multicenter design to reduce any bias in this regard. However, in our study, CCs were examined with a validated and reproducible acquisition protocol (11), reducing the probability of assessment bias. We have little information about patients not eligible for reperfusion therapy, and this restricts our results to a limited population.

Half of our patients were imaged after 6 h from symptoms onset, so our results may be translated also to patients eligible for EVT in the extended time window.

## 5. Conclusion

In conclusion, we did not find any significant clinical or demographic determinant of CCs, except for a negative association with previous cerebrovascular events. Considering the paramount importance of the topic, it is mandatory to go on applying our efforts to this research, which also focuses on other possible factors that may contribute to CC development. Our study generates a hypothesis that deserves to be further explored.

## Data availability statement

The raw data supporting the conclusions of this article will be made available by the authors, without undue reservation.

## Ethics statement

Ethical review and approval was not required for the study on human participants in accordance with the local legislation and institutional requirements. Written informed consent for participation was not required for this study in accordance with the national legislation and the institutional requirements.

## Author contributions

MS: substantially contributed to the acquisition of data, interpretation of data, and drafting of the manuscript. FA: substantially contributed to the analysis, interpretation of data, and critical revision of the manuscript for important intellectual content. AA: substantially contributed to the acquisition of data. GB and EF: substantially contributed to the acquisition and interpretation of data. CS: substantially contributed to the conception and design of the study, interpretation of data, and critical revision of the manuscript for important intellectual content. All the authors provide approval for publication of the content and agree to be accountable for all aspects of the study in ensuring that questions related to the accuracy or integrity of any part of the study are appropriately investigated and resolved.
